# CCL17 and CCL22 chemokines are upregulated in human obesity and play a role in vascular dysfunction

**DOI:** 10.3389/fendo.2023.1154158

**Published:** 2023-04-12

**Authors:** Luisa Hueso, Patrice Marques, Brenda Morant, Herminia Gonzalez-Navarro, Joaquin Ortega, José T. Real, María J Sanz, Laura Piqueras

**Affiliations:** ^1^ INCLIVA Biomedical Research Institute, Valencia, Spain; ^2^ Department of Biochemistry, University of Valencia, Valencia, Spain; ^3^ CIBERDEM: Diabetes and Associated Metabolic Diseases Networking Biomedical Research- Instituto de Salud Carlos III (ISCIII), Madrid, Spain; ^4^ Surgery Service, University Clinic Hospital of Valencia, Valencia, Spain; ^5^ Department of Surgery, University of Valencia, Valencia, Spain; ^6^ Endocrinology and Nutrition Service, University Clinic Hospital of Valencia, Valencia, Spain; ^7^ Department of Pharmacology, University of Valencia, Valencia, Spain

**Keywords:** obesity, adipose tissue, chemokines, inflammation, endothelium, CCL22, CCL17

## Abstract

**Background/Aims:**

Chemokines are known to play critical roles mediating inflammation in many pathophysiological processes. The aim of this study was to investigate the role of chemokine receptor CCR4 and its ligands CCL17 and CCL22 in human morbid obesity.

**Methods:**

Circulating levels of CCL17 and CCL22 were measured in 60 morbidly obese patients (mean age, 45 ± 1 years; body mass index/BMI, 44 ± 1 kg/m^2^) who had undergone bariatric bypass surgery, and 20 control subjects. Paired subcutaneous (SCAT) and visceral adipose tissue (VCAT) from patients were analysed to measure expression of CCR4 and its ligands by RT-PCR, western blot and immunohistochemical analysis. The effects of CCR4 neutralization *ex vivo* on leukocyte-endothelial cells were also evaluated.

**Results:**

Compared with controls, morbidly obese patients presented higher circulating levels of CCL17 (p=0.029) and CCL22 (p<0.001) and this increase was positively correlated with BMI (p=0.013 and p=0.0016), and HOMA-IR Index (p=0.042 and p< 0.001). Upregulation of CCR4, CCL17 and CCL22 expression was detected in VCAT in comparison with SCAT (p<0.05). Using the parallel-plate flow chamber model, blockade of endothelial CCR4 function with the neutralizing antibody anti-CCR4 in morbidly obese patients significantly reduced leucocyte adhesiveness to dysfunctional endothelium, a key event in atherogenesis. Additionally, CCL17 and CCL22 increased activation of the ERK1/2 mitogen-activated protein kinase signalling pathway in human aortic endothelial cells, which was significantly reduced by CCR4 inhibition (p=0.016 and p<0.05).

**Conclusion:**

Based on these findings, pharmacological modulation of the CCR4 axis could represent a new therapeutic approach to prevent adipose tissue dysfunction in obesity.

## Introduction

Obesity is a highly prevalent worldwide health problem characterized by a chronic low-grade inflammatory state (1). In obesity, adipose tissue (AT) and vascular endothelial dysfunction have been reported to increase the risk of suffering cardiovascular disease, insulin resistance and certain types of cancer (2, 3). Excessive fat accumulation dysregulates the microenvironment within adipose depots and systemically alters peripheral tissue metabolism. A growing body of experimental and clinical evidence demonstrates different intrinsic properties between human fat depots, suggesting that visceral AT (VCAT) is the most pathogenic and associated with increased metabolic risk (4). However, the mechanisms that underlie AT heterogeneity remain largely unknown.

Chemokines are small and structurally related disulfide-linked polypeptides which, through their interactions with a family of G protein-coupled receptors, mediate leukocyte chemotaxis and angiogenesis (5, 6). In AT, inflammation is initiated and sustained over time by infiltration of immune cells and dysfunctional adipocytes which secrete adipokines and signal via production of several chemokines (7). The thymus and activation-regulated chemokine (TARC/CCL17) and the macrophage-derived chemokine (MDC/CCL22) are ligands for the CC chemokine receptor 4 (CCR4) and are released by immune cells such as macrophages, dendritic cells, T cells and endothelial cells (8, 9). These chemokines are involved in several cardiovascular and inflammatory diseases such as heart failure (10), asthma (11), type 1 diabetes (12), inflammatory bowel disease (13), and different types of cancer (14–19), yet their role in human obesity remains elusive.

Since these chemokines seem to play a critical role in several pathological inflammatory states, in the present study we hypothesized that peripheral circulating and adipose tissue CCR4 ligands are altered in morbidly obese patients. The aims of our research were to measure circulating CCL17 and CCL22 chemokines and their association with metabolic parameters linked to AT dysfunction. Additionally, we evaluated the expression of CCR4 receptor and its ligands in subcutaneous and visceral fat and investigated the functional consequences of CCR4 blockade in human endothelial dysfunction as well as the signalling pathways underlying these responses.

## Materials and methods

### Study population

We included 60 consecutive obese patients that were selected for Roux-Y- gastric bypass from our Nutrition Section at University Clinic Hospital of Valencia. The group comprised 40 women and 20 men, aged 45 ± 1.5 years, and with a mean body mass index (BMI) of 44.1 ± 0.8 kg/m^2^. Patient selection was based on the recommendations from the National Institute of Health (NIH) for bariatric surgery: BMI > 40 kg/m^2^, or BMI > 30 kg/m^2^ with co-morbidities, who had tried for over 5 years to control their obesity by lifestyle changes and who understood the risks and benefits of the procedure. Exclusion criteria of the study were age < 18 years or > 60 years, pregnancy, and current history of inflammatory, infectious or malignant disease. The study was performed in accordance with the Declaration of Helsinki and ethical approval was obtained from the Research Ethics Committee of the University Clinic Hospital of Valencia. All participants gave written and informed consent.

### Clinical and biochemical data

One month before bariatric surgery, blood samples and anthropometric measures were obtained from all subjects and a complete medical history was collected, including demographics, dietary and smoking habits, cardiovascular risk factors, physical activity and personal and family profile of previous diseases. Inclusion criteria were body mass index (BMI) <30 kg/m^2^, triglycerides <150 mg/dL, total cholesterol <200 mg/L, fasting plasma glucose <100 mg/dL and absence of personal or family history of dyslipidaemia, cardiovascular disease or diabetes. Furthermore, 20 non-obese age-matched, normolipidemic and non-diabetic control subjects were recruited to assess baseline plasma levels.

Between 09:00 and 10:00 a.m. after 10 h overnight fasting, blood samples were collected in heparin tubes from both obese patients and controls. Heparinized whole blood was centrifuged to obtain plasma samples and immediately stored at -80°C. Biochemical parameters including lipid profile, insulin, glucose, and high-sensitivity C-reactive protein were analysed by standard procedures (20). Waist circumference, BMI, systolic and diastolic blood pressure were measured as previously described (21). The homeostasis model assessment (HOMA) [(insulin (mUI/L) × [glucose (mmol/L)/22.5] was used to determine insulin resistance. The same researcher performed all measurements.

### Roux-en-Y gastric bypass

All obese patients subjected to laparoscopic-RYGB were operated by the same surgical team using the procedure previously described (21). Fat samples [paired visceral (VCAT) and subcutaneous adipose tissue (SCAT)] samples were freshly collected during bariatric surgery and immediately placed in cold phosphate-buffered saline (PBS).

### Circulating chemokine measurements

CCL17 and CCL22 were measured using ELISA Quantikine^®^ kits (cat# DY36405 and DY336, R&D Systems, Abingdon, UK) and following the manufacturer’s recommendations and instructions.

### Adipose tissue explant cultures

To measure chemokine secretion, fat explants were dissected into small pieces, washed with Dulbecco´s phosphate -buffered saline (DPBS) at 37°C, and remaining blood cells were removed by centrifugation (400 × g for 1 min). Explants were cultured for 48 h into a 6-well plate with pre-warmed (37°C) endothelial medium (EBM-2; Lonza, Barcelona, Spain) containing 2% bovine serum albumin (BSA), 10% fetal bovine serum (FBS), streptomycin (50 mg/mL), penicillin (50 U/mL), L-glutamine (2 mM). Quantification of CCL17 and CCL22 secretion from AT explants was performed using ELISA kits assays (cat# DY36405 and cat# DY336, R&D Systems, Abingdon, UK).

### Quantitative RT-PCR

Fat samples were homogenised with TRIzol™ Reagent (cat#15596018 Invitrogen, Carlsbard, CA) using a T25 digital ULTRA-TURRAX^®^ (IKA Werke GmbH, Germany) for RNA extraction. For purifying the samples, a RNeasy Lipid Tissue Mini Kit was used (Qiagen, Werfen, Barcelona, Spain). 1 μg of total RNA was reverse transcribed using a TaqMan Reverse Transcription Reagent Kit (ThermoFisher Scientific, Waltham, MA). Expression of CCL22, CCL17 and CCR4 mRNA was detected using human Taqman specific probes from Applied Biosystems: (Hs01574247_m1, Hs00171074_m1 and Hs00747615_s1, respectively). The 2^−ΔΔCt^ method was used for relative quantification of the different transcripts (Applied Biosystems).

### Immunofluorescence analysis

AT samples were fixed and mounted in paraffin and sectioned into 5 μm slides with a microtome (Leica Biosystems, Nussloch, Germany). Antigen was unmasked with proteinase K (cat#S3020, Dako, Santa Clara, CA) and blocked with 15% horse serum for 1 h. Samples were incubated with the following primary antibodies overnight at 4°C: mouse anti-human CCL17 (1:50, cat#DY364-05, R&D Systems), mouse anti-human CCL22 (1:50, cat#DY336, R&D Systems), goat anti-human CCR4 (1:100, ab1669; Abcam, Cambridge, UK), rabbit anti-human CD3 (1:100, cat#C7930, Sigma-Aldrich, St. Louis, MO), rabbit polyclonal anti-human CD31 (1:50, cat#ab32457, Abcam) and rat anti-human Mac-3 (1:100, cat#sc19991, Santa Cruz Biotechnology, Dallas, TX). Alexa-Fluor^®^ 488 goat anti-rabbit (cat#A11034), Alexa-Fluor^®^ 488 donkey anti-rat (cat#A21208), Alexa-Fluor^®^ 594 goat anti-rat (cat#A1107), Alexa-Fluor^®^ 594 goat anti-mouse (cat#A1105) and Alexa-Fluor^®^ 594 chicken anti-goat (cat#A21468) antibodies were used as secondary antibodies (dilution 1:1000; all from Molecular Probes, Eugene, OR). Nuclei were stained with Hoechst (1:4000). Afterwards, five fields from each section were captured with a Zeiss Axio Observer A1 fluorescence microscope (Carl Zeiss Micro Imaging GmbH, Oberkochen, Germany).

### Human aortic endothelial cells culture

Human aortic endothelial cells (HAEC, cat#CC-2535, Lonza, Barcelona, Spain) were cultured and maintained in endothelial medium (EBM-2) containing L-glutamine (2 mM), 2% bovine serum albumin (BSA), 10% fetal bovine serum (FBS), penicillin (50 U/mL) and streptomycin (50 mg/mL).

### Endothelial proliferation assay

HAEC were incubated with plasma (diluted 1:10 in HBSS) from morbidly obese patients or control subjects for 24 h. Some plates were preincubated with a monoclonal neutralizing antibody against human CCR4 receptor (3 μg/mL, Mogamulizumab, Absolute antibody, UK) or with an isotype-matched control antibody for 30 min. To determine endothelial cell proliferation cells were incubated with bromodeoxyuridine (BrdU) incorporation (50 μM), as previously described (22). Cells were fixed with 4% paraformaldehyde for 20 min, permeabilized with 1.0% Triton X-100/PBS for 15 min and incubated with PBS containing 10% normal goat serum for 1 h at room temperature. BrdU-positive cells were detected by immunofluorescence with an anti-BrdU Alexa Fluor-488 antibody (B35130, Invitrogen). Nuclei were visualized with Hoechst staining (1:4000). Next, images were captured with a Zeiss Axio Observer A1 fluorescence microscope (Carl Zeiss Micro Imaging GmbH) under 40x magnification. Cell proliferation was determined as the percentage of BrdU positive cells relative to total cell count in 3 randomly selected fields (40x).

### Endothelial differentiation assay

Matrigel Growth Factor Reduced Basement Membrane Matrix (150 µL, cat#354230, Corning, NY, USA) was added to 48-well tissue culture plates and polymerized for 45 minutes at 37°C as described (22). HAEC (15x10^3^ cells/well) were seeded into Matrigel in EBM-2 without growth factor medium containing 1% FBS. Cells were preincubated for 30 min with a monoclonal neutralizing antibody against human CCR4 receptor (3 μg/mL) or with an isotype-matched control antibody. Next, cells were incubated with diluted serum (1:10 in HBSS) from morbid obese patients and controls for 4 h. Afterwards, phase contrast micrographs (Axio Observer A1, Carl Zeiss) were recorded, and the number of tubes was determined in 4 random fields (10x).

### Leukocyte-endothelial cell interactions under flow conditions

To determine leukocyte-endothelial cell interactions *ex vivo* a dynamic flow chamber assay was used as previously described (23). First, HAEC were incubated with TNFα (20 ng/mL) or vehicle for 24 h. Additionally, some plates were preincubated with a neutralizing monoclonal antibody against human CCR4 receptor (3 μg/mL) or with an isotype-matched control antibody added 30 min before blood perfusion. The flow chamber (GlycoTech, Rockville, MD) was assembled and placed onto an inverted microscope stage. Next, diluted blood (1:10 in HBSS) from obese patients and controls was perfused across the endothelial cell monolayers and cell interactions were determined after 5 min of perfusion at 0.5 dyn/cm^2^ and visualized with an inverted microscope (Zeiss Axio Observer A1 microscope, Thornwood, NY). After 5 minutes of perfusion, 6 random fields were recorded for 10 seconds each using a VideoLab software (Ed Marcus Lbs, Newton, MA). Leukocytes were manually counted and the total number of interacting cells on the surface of endothelial cells was quantified as adherent (cells that remained stationary for the 10-second observation period) as described in the literature (24).

### Western blotting

Homogenates from endothelial cells or fat explants were sonicated and centrifuged (20 min, 4°C, 7000 g). Protein quantification was performed by using bicinchoninic acid assay kit (ThermoFisher). Then, laemmli sample buffer was added to samples and were heated at 95°C for 5 min. Samples were separated by SDS-PAGE and transferred to nitrocellulose membranes, which were then blocked in 5% BSA/Tris-buffered saline containing 0.1% Tween. Membranes were incubated overnight at 4°C with primary antibodies against human CCR4 (1:200, cat#NB100-56336, Novus Biologicals, Centennial, CO), rabbit anti-human phospho-p44/42 MAPK (ERK1/2) (1:500, cat#4377, Cell Signalling, Danvers, MA), rabbit anti-human p44/42 MAPK (ERK1/2) (1:500, cat#4695, Cell Signalling) and mouse monoclonal antibody against human β-actin (1:1000, cat# SAB1305546, Sigma-Aldrich). Next, membranes were washed, and incubated with secondary antibodies (1:2000, cat#P044, or cat#P0447, Dako) for 1 h. Membranes were developed using the ECL procedure (GE Healthcare) and signals were recorded using luminescent analyser (ImageQuant LAS500, GE Healthcare). ImageJ software (NIH) was used for densitometry analysis.

### Statistical analysis

Data were considered statistically significant at p<0.05. To analyse the correlation of CCL17 and CCL22 with clinical variables the Spearman correlation test was applied. Kolmogorov-Smirnov test was used to assess normality of distribution of data. Two-tailed Student´s t test (Wilcoxon matched-pair signed-rank test) was used to compare data of two groups that passed both normality (Kolmogorov-Smirnov test) and equality of variance (Levene´s test); if not, the non-parametric Mann Whitney U test was performed. For comparisons among multiple groups that passed both normality and equality of variance, we used ANOVA (one-way analysis of variance) followed by *post hoc* analysis (Bonferroni test); otherwise, the non-parametric Kruskal–Wallis test followed by Dunn´s *post hoc* analysis was used.

## Results

### CCL17 and CCL22 circulating levels are elevated in patients with morbid obesity

**Table 1** summarizes the biochemical and clinical characteristics from 60 patients with morbid obesity and 20 controls.

**Table 1:** Demographic and clinical parameters of morbidly obese patients and control subjects.

	Morbid obese patients	Controls	
	(n=60)	(n=20)	p value
**Age, years**	45.04 ± 1.5	44.15 ± 1.4	0.7621
**Female sex,** % (n)	66.7 (40)	60 (12)	
**BMI** (Kg/m^2^)	44.1 ± 0.8	25.6 ± 0.8	<0.0001
**Weight** (Kg)	121.7 ± 2.9	71.5 ± 3.0	<0.0001
**Waist circumference** (cm)	128.3 ± 1.9	80.9 ± 2.8	<0.0001
**Fasting glucose** (mg/dL)	96.3 ± 3.2	90.1 ± 3.0	0.2919
**Plasma insulin** (mlU/mL)	20.4 ± 1.5	8.6 ± 1.0	<0.0001
**HOMA-IR index**	5.1 ± 0.5	2.0± 0.3	0.0015
**Triglycerides** (mg/dL)	107.5 ± 5.6	68.1 ± 4.2	<0.0001
**Total cholesterol** (mg/dL)	150.7 ± 4.0	164 ± 8.2	0.1981
**LDL cholesterol** (mg/dL)	103 ± 3.6	111 ± 1.7	0.126
**HDL cholesterol** (mg/dL)	40.3 ± 1.1	42.9 ± 1.6	0.081
**CRP** (mg/dL)	9.2 ± 1.0	1.7 ± 0.2	0.0047
**Systolic blood pressure** (mmHg)	131.5 ± 2.1	117.3 ± 3.4	0.0008
**Diastolic blood pressure** (mmHg)	83.0 ± 1.2	70.9 ± 2.2	<0.0001
**Diabetes,** % (n)	45 (27)	–	–
**Hypertension,** %(n)	36.7 (22)	–	–

BMI, body mass index; LDL, Low-density lipoprotein; HDL, High-density lipoprotein; CRP, high sensitivity C-reactive protein.

**Figure 1:**
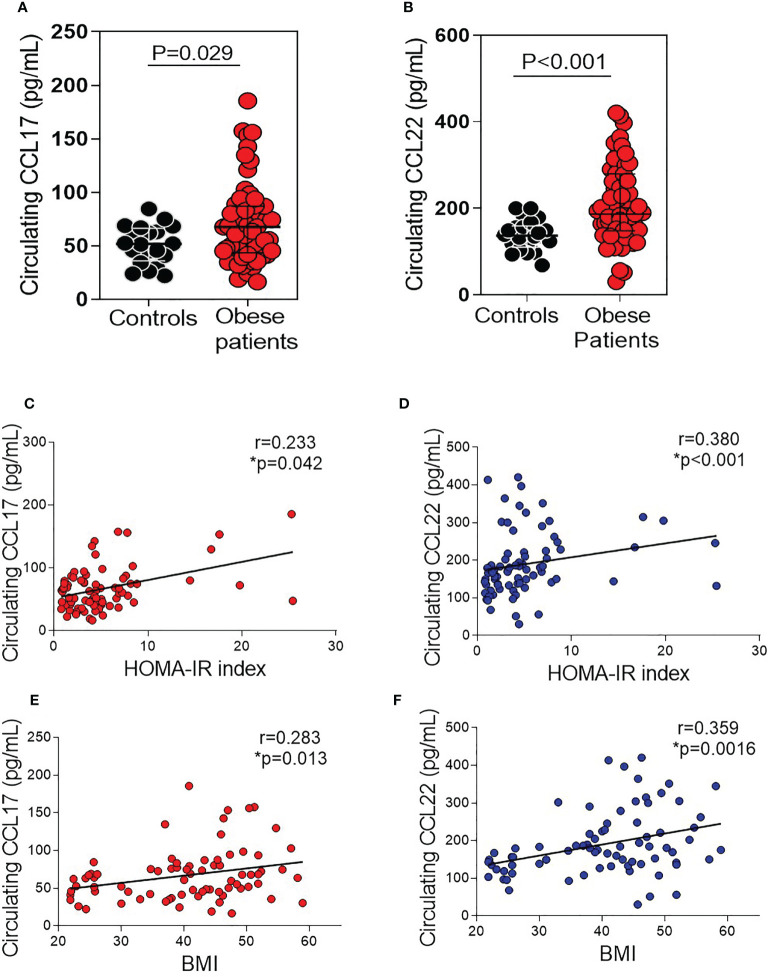
Circulating CCL17 and CCL22 chemokine levels in morbidly obese patients and age-matched controls. **(A)** CCL17 and **(B)** CCL22 levels were measured in plasma samples from obese patients (n = 60) and controls (n = 20). Scatter dot plots showing median with interquartile range. Comparison between groups were made by Mann Whitney test. Spearman test shows a positive correlation between CCL17 and CCL22 with HOMA-IR Index **(C, D)** and BMI **(E, F)** (n = 20 control subjects and n = 60 morbidly obese patients).

Plasma levels of CCL17 were notably elevated in patients with morbid obesity (median, 67.8 pg/mL, range 16.6–185.6 pg/ml) compared with control subjects (median 51.8 pg/mL, range 22.2–84.5 pg/mL, p=0.029) (**Figure 1A**). Similarly, patients with morbid obesity showed systemic CCL22 levels higher than controls (median 186.6 pg/mL, range 30.3–420.8 pg/mL in patients *vs.* median 136.8 pg/mL, range 68.2–200.3 pg/mL in controls, p<0.001) (**Figure 1B**). We next tested whether the changes in CCL17 and CCL22 plasma levels were associated with clinical parameters. Spearman correlation analysis showed a positive correlation of circulating CCL17 and CCL22 levels with HOMA-IR index (r=0.233, p=0.042 and r=0.38, p <0.001, respectively, **Figures 1C, D**), and with BMI (r=0.283, p=0.013 and r=0.359, p=0.0016, respectively (**Figures 1E, F**).

### CCL17 and CCL22 expression and release are increased in visceral AT compared with subcutaneous AT from patients with morbid obesity

**Figure 2:**
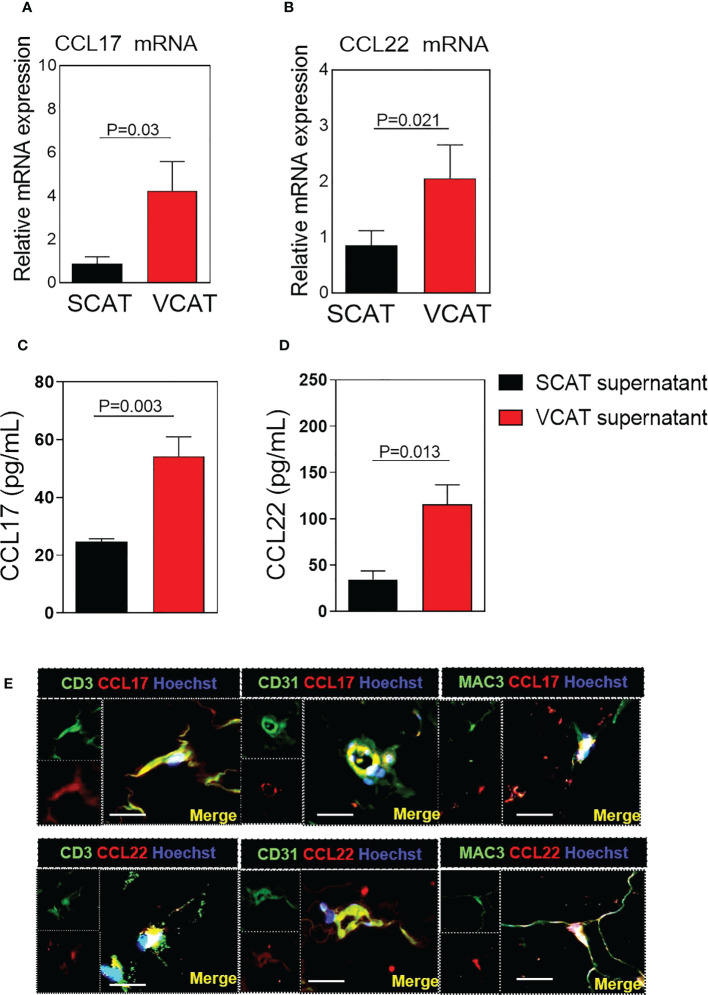
Expression of CCL17 and CCL22 is increased in VCAT from morbidly obese patients. Relative quantification of mRNA levels for **(A)** CCL17 and **(B)** CCL22. Comparisons between groups were made by Wilcoxon matched-pair signed-rank test. Values are expressed as mean ± SEM (n = 33). **(C)** CCL17 and **(D)** CCL22 chemokine release into conditioned media was determined after 48 h of SCAT and VCAT explant culture. Chemokine secretion is expressed as pg/ml in the supernatant. Values are expressed as mean ± SEM (n = 22). Comparison between groups were made by Mann Whitney test. **(E)** Immunofluorescence representative images showing colocalization of CCL17 with CD3 (lymphocytes), CD31 (endothelial cells) and Mac-3 (macrophages); or CCL22 with CD3, CD31, Mac-3 in VCAT. Immunoreactivity was visualized using Alexa Fluor 594 (CCL17 and CCL22, *red*) and Alexa Fluor 488 (CD31, CD3, Mac-3, *green*) secondary antibodies. Nuclei were stained with Hoechst (*blue*). Scale bar, 20 μm. Nuclei were stained with Hoechst (*blue*).

**Figure 3:**
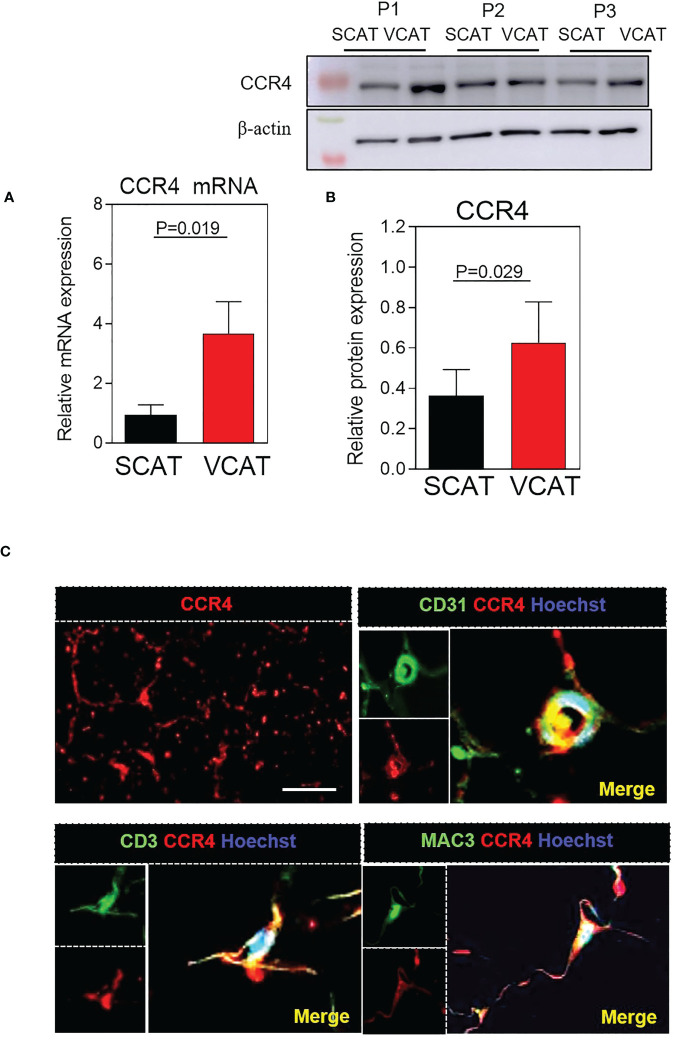
CCR4 expression is increased in VCAT from morbid obese patients. **(A)** Relative quantification of CCR4 mRNA levels. Values are expressed as mean ± SEM (n = 33); **(B)** Western blot analysis of CCR4 relative protein expression to β-actin in paired and SCAT and VCAT samples. Representative western blots are shown from three different patients (P1–3). Data represent the mean ± SEM of protein densitometry. Comparison between groups were made by Wilcoxon matched-pair signed-rank test. **(C)** Immunofluorescence representative images showing CCR4 expression in VCAT. Colocalization of CCR4 with CD3 (lymphocytes), CD31 (endothelial cells) and Mac-3 (macrophages) in VCAT. Immunoreactivity was visualized using Alexa Fluor 594 (CCR4, *red)* and Alexa Fluor 488 (CD31, CD3, Mac-3, *green*) secondary antibodies. Scale bar, 50 μm. Nuclei were stained with Hoechst (*blue*).

Because the upregulation of circulating CCR4 ligands in the plasma of patients with morbid obesity, their expression was evaluated in paired VCAT and SCAT obtained through bariatric surgery in these subjects. As shown in **Figure 2**, both CCL17 mRNA (p=0.03, **Figure 2A**) and CCL22 mRNA (p=0.021, **Figure 2B**) expression were notably higher in visceral compared with subcutaneous AT. We further measured CCL17 and CCL22 release in the secretome of AT cultured explants. Secretion of both CCL17 and CCL22 chemokines was significantly higher in VCAT than in SCAT supernatants (p<0.05, **Figures 2C, D**). Next, by double labelling immunofluorescence analysis we detected CCL17 and CCL22 expression mainly in CD3+ lymphocytes, CD31+ microvessels and Mac3+ macrophages (**Figure 2E**) from AT explants. Additionally, we analyzed their chemokine receptor CCR4 gene expression in fat depots from obese subjects. As shown in **Figure 3A**, CCR4 mRNA expression was significantly higher in VCAT than in SCAT (p=0.019, **Figure 3A**). To corroborate these observations, analysis of CCR4 protein expression was carried out in fat explants from patients. As illustrated in **Figure 3B**, a marked upregulation of CCR4 was found in VCAT compared to SCAT (p=0.029, **Figure 3B**). Double labelling immunofluorescence analysis showed that CCR4 is mainly expressed in lymphocytes (CD3+), endothelial cells (CD31+), and macrophages (Mac3+) in VCAT (**Figure 3C**).

### Enhanced leukocyte adhesiveness in endothelial cell adhesion found in patients with morbid obesity was reduced by functional CCR4 blockade

**Figure 4:**
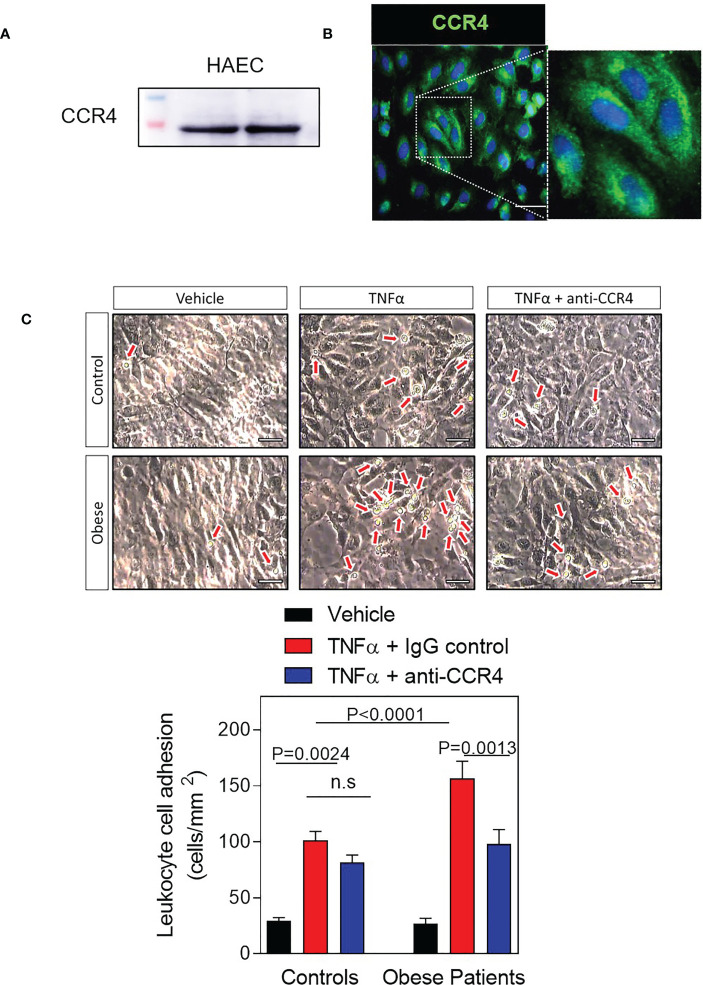
*Ex vivo* blockade of CCR4 receptor decreases TNFα-induced leukocyte-endothelial cell adhesion in obese patients. **(A)** Protein CCR4 expression in human aortic endothelial cells (HAEC) **(B)** Immunofluorescence of representative images showing CCR4 expression in HAEC. Scale bar, 50 μm. **(C)** Confluent HAECs were stimulated or not with TNFα (20 ng/mL) for 24 h. In parallel, some samples were preincubated with a monoclonal neutralizing antibody against human CCR4 receptor (3 μg/mL). Diluted heparinized whole blood (1:10) from patients (n=16) or healthy controls (n=9) was perfused across endothelial cells monolayers for 5 min at 0.5 dyn/cm^2^. Values are expressed as mean ± SEM. Representative images show adhered leukocytes to endothelial cell monolayer. Scale bar, 100 μm. Comparisons between groups were made with 1-way ANOVA.

Our next step was to study CCR4 expression and function on endothelial cells. First, immunoblotting of cell lysates revealed CCR4 receptor expression in human aortic endothelial cells (HAEC) (**Figure 4A**). These observations were confirmed by immunofluorescence analysis: CCR4 expression was detected on the endothelial cell surface (**Figure 4B**).

Moreover, obesity accelerates the development of vascular endothelial dysfunction, the first stage of atherogenesis, which is characterized by an enhanced leukocyte adhesion to the endothelium and a subsequent migration of these cells into the arterial subendothelial region (23, 25). Hence, we next evaluated leukocyte–endothelial cell interactions *ex vivo* under dynamic flow conditions (**Figure 4C**). To carry out these assays, HAEC plates were stimulated with TNFα (20 ng/mL) for 24 hours to simulate a dysfunctional endothelium, and then heparinized blood from obese patients or healthy controls was perfused across endothelial cells monolayers to analyse leukocyte adherence. As shown in **Figure 4C**, we detected that pre-stimulated HAEC monolayers with TNFα and the perfused with the diluted blood, increased leukocyte adhesion compared to vehicle in both groups but was significantly greater for patients with morbid obesity (p<0.0001, **Figure 4C**). Of note, neutralizing CCR4 activity on the endothelial cell surface resulted in a significant decrease in TNFα-stimulated leukocyte adhesion when blood from obese patients was perfused (p=0.0013, **Figure 4C**) but not when it was from controls, indicating that CCR4 axes play an important role in leukocyte recruitment and endothelial impairment in patients with morbid obesity.

**Figure 5:**
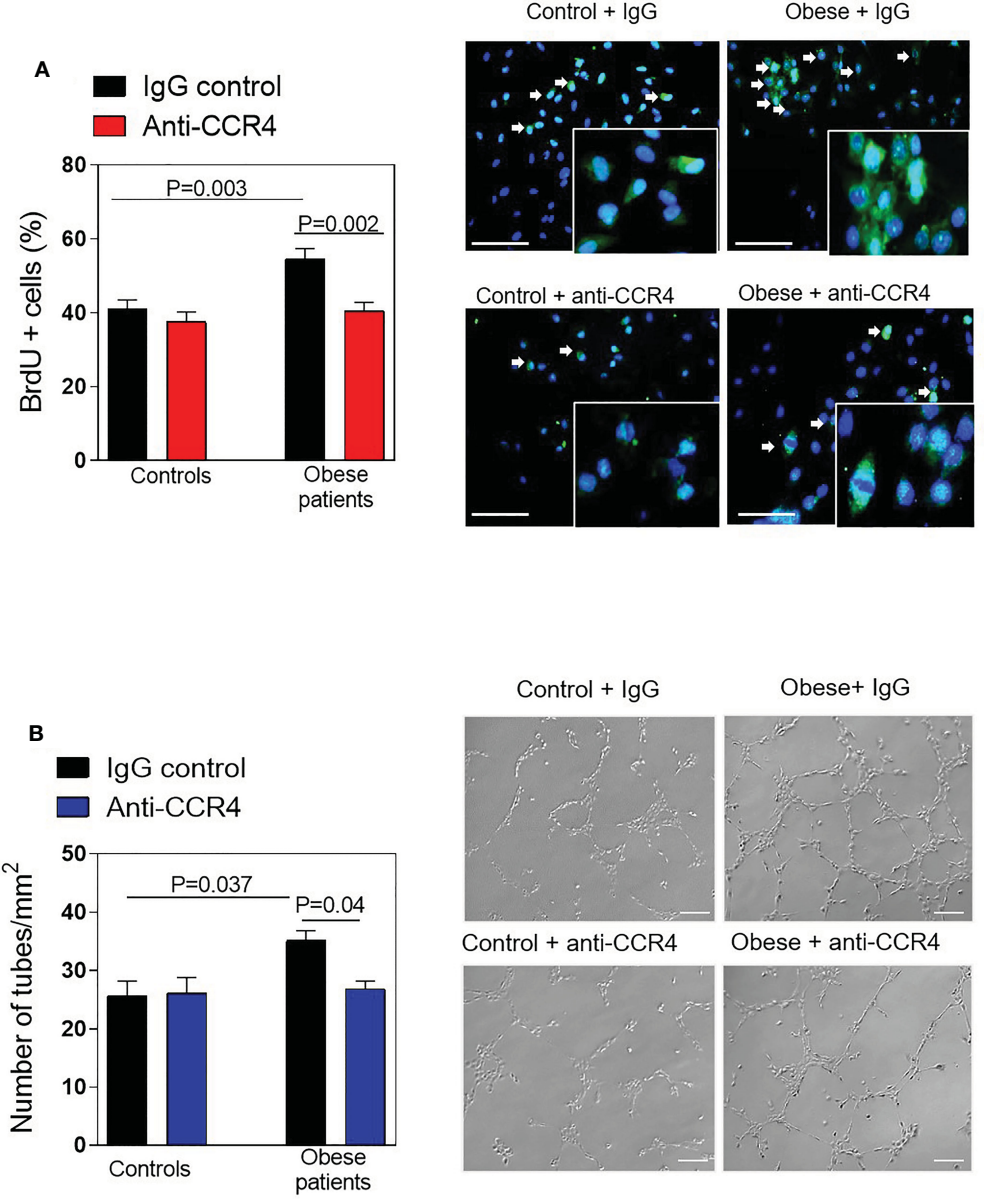
Neutralizing CCR4 reduces endothelial cell proliferation and morphogenesis. **(A)** HAEC were incubated with plasma (diluted 1:10 in HBSS) from morbid obese patients or controls subjects for 24h. Some plates were preincubated with a monoclonal blocking antibody against human CCR4 (3 µg/mL). Percentage of proliferating endothelial cells was analysed by BrdU incorporation. Data represent the mean ± SEM of the percentage of BrdU+ cells in 3 random fields (40x). (n = 8 control subjects and n = 8 morbidly obese patients). Right panels show representative images of endothelial cell proliferation. Scale bar, 100 μm. **(B)** Cells cultured in Matrigel were incubated with plasma (diluted 1:10 in HBSS) from obese patients or controls. Phase contrast images were taken after 4 h of incubation and the number of tube-like structures were quantified. Data represent mean ± SEM of the number of tube-like structures or total length of the tubes in 4 random fields (10x). (n = 10 control subjects and n = 10 morbidly obese patients). Right panels show representative images of endothelial cell differentiation on Matrigel. Scale bar, 200 μm.

Based on the above observations, functional studies *ex vivo* were performed using the plasma of obese patients in HAEC to examine the effect of CCR4 blockade. First, in the presence of an irrelevant antibody, endothelial cells incubated with diluted plasma from obese patients displayed higher proliferation than plasma from control subjects (p=0.003 **Figure 5A**). This was significantly reduced by CCR4 blockade (p=0.002, **Figure 5A**). Similarly, a substantial increase in endothelial differentiation was observed when endothelial cells were incubated with plasma from obese subjects compared to plasma from controls (p=0.037, **Figure 5A**), and neutralization of CCR4 resulted in a significant decrease of cell morphogenesis/tubulogenesis (p=0.04, **Figure 5B**) induced by plasma from obese patients.

### CCL17 and CCL22 activate the ERK1/2 MAPK signaling pathway in AT.

**Figure 6:**
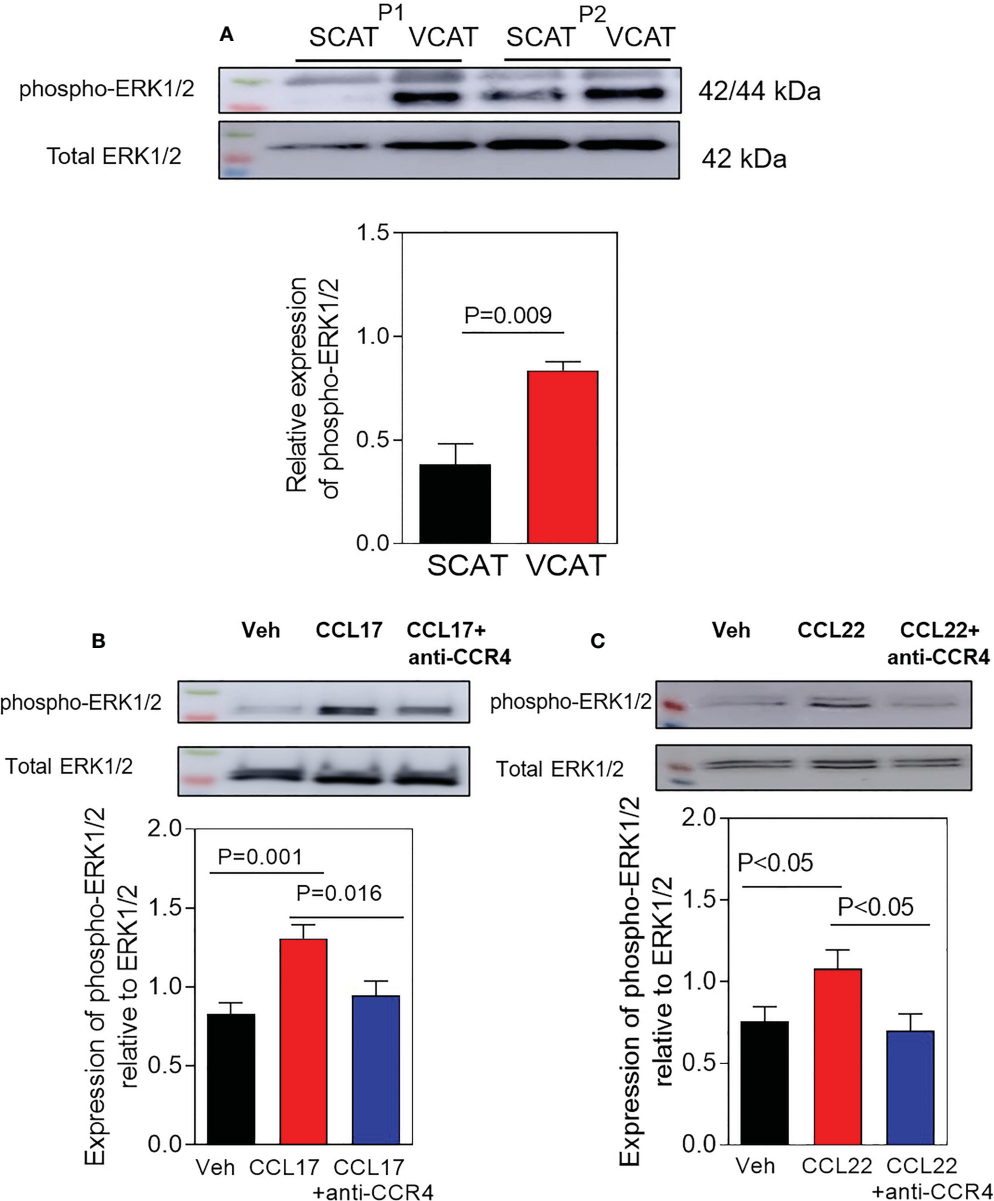
Activation of phospho-ERK1/2 MAPK signaling in VCAT from morbid obese patients. **(A)** Western blot analysis of phospho-ERK1/2 MAPK in paired SCAT and VCAT samples from morbid obese patients (P1-2). Representative Western blot is shown. Data represent the mean ± SEM of protein densitometry (n = 6). Comparisons between groups were made by two-tailed Student´s test. In additional experiments, HAEC were incubated with vehicle **(B)**, hrCCL17, or **(C)** hrCCL22 (10 ng/ml) for 30 minutes. Some cells were pre-treated with a mouse monoclonal blocking antibody against human CCR4 (3 µg/ml) during 10 minutes before treatment. Representative Western blot is shown. Data represent the mean ± SEM of protein densitometry (n = 7). Comparison between groups were made by one-way ANOVA test.

Extracellular signal-regulated kinase 1/2 (ERK1/2) is a serine/threonine protein kinase belonging to the mitogen-activated protein kinase (MAPK) which mediates many aspects of cell proliferation, differentiation, and vascular inflammation (26). Then, we studied ERK1/2 phosphorylation in subcutaneous and omental AT depots from patients with morbid obesity. Western blot analysis revealed that basal phosphorylation levels of ERK1/2 were significantly higher in visceral AT from obese patients (**Figure 6A**, p=0.009) than in subcutaneous AT. We further characterized this signalling pathway in the presence of CCL17 or CCL22 in human aortic endothelial cells. This analysis revealed increased phosphorylation of the ERK1/2 pathway when cells were pre-incubated with hr-CCL17 (p=0.001, **Figure 6B**) or hr-CCL22 (p<0.05, **Figure 6C**) and significant reductions were detected when cells were incubated with a CCR4 neutralizing antibody (p=0.016 and p<0.05, **Figures 6B, C**).

## Discussion

In obesity, chronic inflammation is considered a significant risk factor for developing type 2 diabetes, cardiovascular diseases and cancer (27). Here we report that compared with in control subjects, circulating chemokines CCL17 and CCL22 are significantly augmented in patients with morbid obesity and positively correlated with BMI and insulin resistance.

During obesity, there is a chronic low-grade inflammation associated to adipose tissue dysfunction which accelerates the onset of insulin resistance and diabetes. Infiltrating immune cells produce several factors, such as cytokines/chemokines, which participate in adipose tissue remodeling and cell signaling (28, 29). It is well known that human visceral and subcutaneous fat depots have significantly different properties, this makes visceral adipose tissue a more pathogenic depot linked to higher metabolic risk (30–32); however, the underlying mechanisms have not yet been completely elucidated. Here, we showed that visceral fat depots expressed and secreted CCL17 and CCL22 to a higher degree than subcutaneous fat from obese patients. Interestingly, the upregulation of these chemokines correlated with a higher expression of their receptor CCR4 in T-lymphocytes, endothelial cells and macrophages in visceral fat, suggesting that these mediators might promote inflammation during AT expansion through autocrine and/or paracrine effects, resulting in metabolic dysfunction in obese patients.

Excess adiposity contributes to endothelial dysfunction, one of the earliest stages of atherogenesis. This endothelium phenotype, which is proinflammatory and prothrombotic, induces leukocyte extravasation into the subendothelial space (33). Associations between endothelial dysfunction and inflammation have been described in patients with obesity (28, 34). Given that little is known regarding CCR4 axes and leukocyte-endothelium interactions in human morbid obesity, we sought also to study their functional significance in an *ex vivo* model of dysfunctional endothelium using the parallel-plate flow chamber system. We observed that leukocyte adhesion to human endothelial cells stimulated with TNFα was significantly higher in the morbid obesity than in the control group. Our findings suggest that CCR4 is essential for leukocyte adherence to injured vascular endothelium because functional blocking of this receptor lowers leukocyte adhesion to levels found in the controls. Our results of elevated CCL17 and CCL22 levels in peripheral blood from morbidly obese patients suggest that these chemokines may play a role in leukocyte trafficking and endothelial dysfunction in obesity, in addition to their role as chemoattractants in the adipose tissue milieu (21).

Angiogenesis, the excessive production of new blood vessel capillaries, is associated to chronic inflammatory processes, including atherosclerotic plaque progression/destabilization and tumor growth (35). Obesity is linked with an upregulation of angiogenic factors such as vascular endothelial growth factor (VEGF) (36, 37), which have been correlated with metabolic dysfunction (37, 38) and tumor development (35, 39). Some of those angiogenic players are therefore expressed in AT depots, and beside their local effects in AT, they might have deleterious effects in other capillary beds and contribute to endothelial dysfunction and cancer development (40, 41). CCR4 and its ligands CCL17 and CCL22 have been shown to mediate stimulatory signals for angiogenesis and tumor growth, and CCR4 blockade was associated with reduced tumor invasion and metastasis and better prognosis in some human cancer types such as hepatoma and colon cancer (15, 42). In our study, we observed potential beneficial effects of CCR4 blockade in the context of obesity, since treatment with a neutralizing antibody against CCR4 led to a decrease of endothelial cell proliferation and differentiation. Future *in vivo* animal studies will be necessary to substantiate the mechanisms of these effects in the context of obesity.

ERK1/2 signaling activation is involved in multiple downstream pathways associated to the production of cytokines/chemokines, angiogenesis and endothelial cell proliferation (43). ERK1/2 signalling has been observed in metabolic alterations such as diabetes (44) and obesity related-vascular inflammation (45). In agreement with this, we detected an increased basal phosphorylation of ERK1/2 in visceral fat of morbidly obese patients. Furthermore, from an *in vitro* setting, we observed that human recombinant CCL17 and CCL22 increased ERK1/2 activation in endothelial cells, which was reverted by CCR4 blockade. Taken together, our findings strongly suggest that the ERK1/2 signaling pathway could be involved in CCL17 and CCL22 effects in obesity-related endothelial dysfunction.

Some limitations of the present study should be underlined, mainly the non-availability of AT from non-obese subjects, which precludes the study of CCR4 in adipose tissue of lean subjects. Additionally, most of our functional studies were performed *ex vivo*, therefore we cannot exclude that other chemokines/cytokines may contribute to the deleterious effects observed in these patients. Future *in vivo* studies in an animal model of obesity are required to delineate the potential benefit of altering the CCR4/CCL17/CCL22 signalling *in vivo* and assess the therapeutic window of CCR4 inhibition. It is also necessary to replicate these results in larger groups of obese people in order to assess the significance of CCR4 in mediating vascular dysfunction and its connection to metabolic complications associated with obesity.

In conclusion, our data suggest that the CCR4/CCL17-CCL22 axis might be involved in metabolic and vascular alterations associated with human morbid obesity, and that its pharmacological modulation may represent a promising target for the treatment of obesity-related cardiovascular and metabolic complications.

## Data availability statement

The raw data supporting the conclusions of this article will be made available by the authors, without undue reservation.

## Ethics statement

The studies involving human participants were reviewed and approved by Research Ethics Committee of the University Clinic Hospital of Valencia. The patients/participants provided their written informed consent to participate in this study.

## Author contributions

LH, PM, BM: Development of experiments, preparation of the figures, analysis of the data. LP, M-JS, JR, JO: Conception, design and interpretation of the data, drafting of the manuscript, resources, review & editing. All authors have read and approved the submitted version of the manuscript.
