# Supplementary material for: CCL17 and CCL22 chemokines are upregulated in human obesity and play a role in vascular dysfunction
Source: Front Endocrinol (Lausanne). 2023 Apr 12;14:1154158. doi: 10.3389/fendo.2023.1154158 (PMC10130371; doi:10.3389/fendo.2023.1154158)
Supplement: Supplementary file 1 [file DataSheet_1.pdf]

Supplementary Material

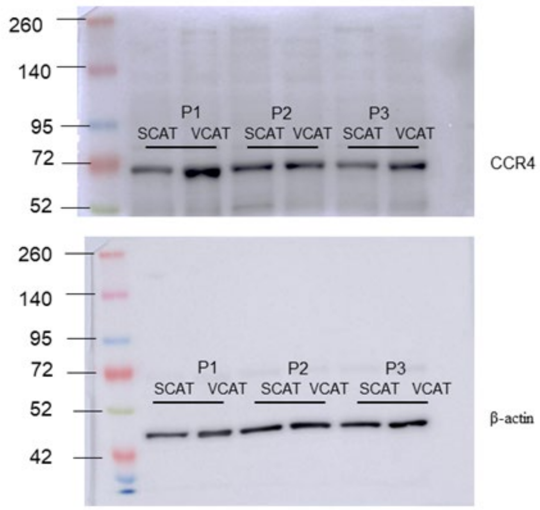

Supplementary Figure 3B. Western blot analysis of CCR4 relative protein expression to β-actin in paired and SCAT and VCAT samples. Representative western blots are shown from three different patients (P1–3). Membranes with the protein molecular weight marker are showed.

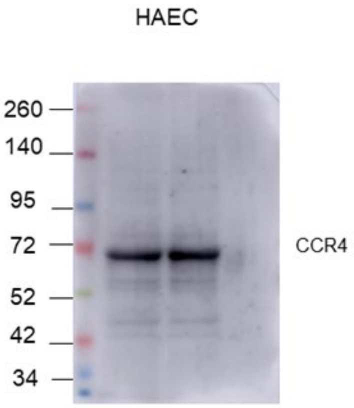

Supplementary Figure 4B. Western blot analysis of CCR4 relative protein expression to in human aortic endothelial cells (HAEC). Membranes with the protein molecular weight marker are showed.

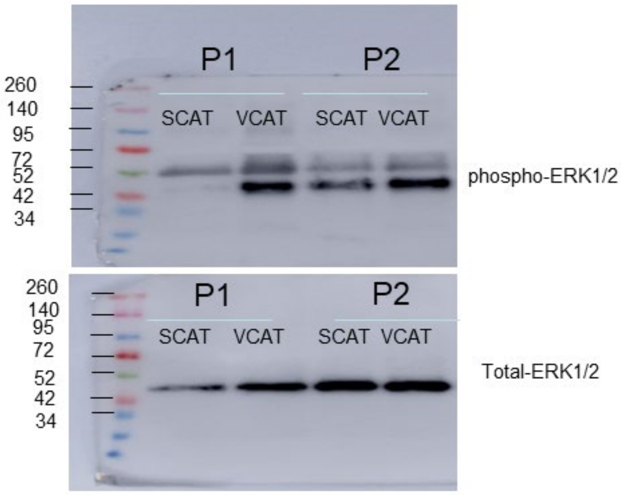

Supplementary Figure 6A. Western blot analysis of phospho-ERK1/2 MAPK in paired SCAT and VCAT samples from morbid obese patients (P1-2). Membranes with the protein molecular weight marker are showed.

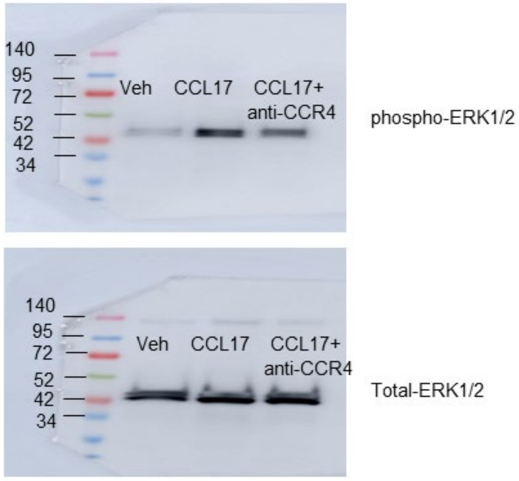

Supplementary Figure 6B. Western blot analysis of phospho-ERK1/2 MAPK. HAEC were incubated with vehicle hrCCL17(10 ng/ml) for 30 minutes. Some cells were pre-treated with a mouse monoclonal blocking antibody against human CCR4 (3 µg/ml) during 10 minutes before treatment. Membranes with the protein molecular weight marker are showed.

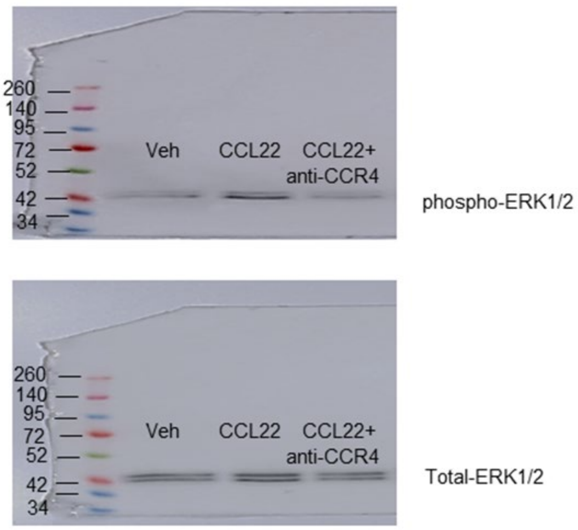

Supplementary Figure 6C. Western blot analysis of phospho-ERK1/2 MAPK. HAEC were incubated with vehicle hrCCL22(10 ng/ml) for 30 minutes. Some cells were pre-treated with a mouse monoclonal blocking antibody against human CCR4 (3 µg/ml) during 10 minutes before treatment. Membranes with the protein molecular weight marker are showed.
